# Deep learning approaches for head pose estimation in sports impacts

**DOI:** 10.1007/s12283-026-00552-9

**Published:** 2026-05-21

**Authors:** Thomas Aston, Georgios Machtsiras, Filipe Teixeira-Dias

**Affiliations:** 1 Institute for Infrastructure and Environment (IIE), School of Engineering, The University of Edinburgh, Edinburgh, UK; 2 Institute for Sport, Physical Education and Health Sciences (ISPEHS), Moray House School of Education and Sport, The University of Edinburgh, Edinburgh, UK

**Keywords:** Computer vision, Motion capture, Head impact, Validation

## Abstract

Videogrammetry can quantify head acceleration events in sport, but because standard datasets lack the large rotations, rapid motion, and frequent occlusion characteristic of sports collisions, the accuracy of modern deep learning pose estimators in this context remains unclear. This study addresses this gap by benchmarking three models for monocular head pose estimation during controlled football headers: a direct head pose regressor, an end-to-end face reconstruction model, and a full-body human mesh recovery model. Ten participants performed linear and rotational headers. Synchronised 1000 Hz infrared motion capture provided ground-truth orientations, while dual 50 Hz video cameras supplied frontal and side views. Model outputs from standardised detections were temporally smoothed and evaluated using geodesic and incremental geodesic error metrics. All models achieved single-digit mean geodesic (4°–8°) and incremental geodesic (<4°) errors. SAM 3D yielded the lowest mean errors (4.59° and 1.99°, respectively) and showed lower sensitivity to occlusion and temporal impact phase. By uniquely comparing head-only and full-body approaches, results demonstrate that modern full-body human mesh recovery models outperform dedicated head pose estimators under the heavy occlusion and dynamic conditions typical of sports collisions. Errors increased for side-view footage, rotational trials, and low facial visibility. These findings support using deep learning, particularly full-body mesh recovery, for semi-automated videogrammetric reconstruction of head acceleration events.

## Introduction

The collision-intensive nature of contact sports exposes athletes to frequent head acceleration events (HAEs) [1, 2], with growing concern around their contribution to sports-related concussion [3, 4] and increased neurodegenerative disease risk [5–9]. Quantifying the biomechanical load associated with these events is therefore a priority [10]. Videogrammetric approaches offer a scalable, non-invasive way to estimate impact speeds [11–13], orientations [14], and body pose during collisions [15, 16], supporting physical and computational reconstruction efforts [17, 18], but widespread adoption remains limited [19].

Early videogrammetric studies relied on manual or semi-automated point tracking to estimate translational motion [13, 20, 21], with head orientation typically approximated downstream. Model-based image matching (MBIM) [22] overcomes this by aligning a 3D model to each frame to directly estimate rotation, and has been applied in American football [12, 23–25], rugby [26], cricket [14], and skiing [27]. However, MBIM remains labour intensive, with over 60 h reportedly required to reconstruct a single rugby HAE [26].

MBIM aligns closely with the head pose estimation problem in computer vision, now commonly tackled using deep learning. State-of-the-art approaches either directly regress orientation from cropped images [28] or infer it by reconstructing a 3D morphable face model (3DMM) [29, 30]. In parallel, human mesh recovery (HMR) models estimate whole-body pose and shape using parametric bodies such as SMPL [31] or the Momentum Human Rig (MHR) [32], implicitly yielding head orientation and potentially leveraging whole-body context during occlusion [33–35].

Application of such models to sports HAEs remains limited. Prior work has used pose estimations from skiing falls [15] and cycling crashes [36], but the accuracy of modern deep learning models for estimating head orientation from a single camera during HAEs (a key parameter in biomechanical reconstruction) has not been systematically assessed. Standard head pose benchmarks such as BIWI and CMU-Panoptic [37, 38] involve slow, controlled motion with minimal occlusion and therefore do not accurately reflect HAE conditions.

This study therefore addresses this gap by uniquely benchmarking and comparing the rotational accuracy of three deep learning models for monocular head pose estimation under sports-relevant conditions: a wide-yaw regression model (6DRepNet360 [39]), a 3DMM-based head pose estimator (VGGHeads [30]), and a full-body HMR model (SAM 3D Body [35]). Performance is evaluated on a laboratory dataset of football headers featuring large rotations, rapid motion, and frequent occlusion, demonstrating that full-body HMR approaches can overcome the limitations of dedicated head pose estimators in these challenging scenarios.

## Methods

### Experimental data

Reference head pose (ground-truth) and synchronised RGB video were obtained during a laboratory football heading protocol. All procedures satisfactorily completed the Research Ethics and Integrity Self-Assessment process at The University of Edinburgh’s School of Engineering, in accordance with UK Research Integrity Office (UKRIO) guidelines; participants were informed about study procedures and provided written consent.

**Fig. 1:**
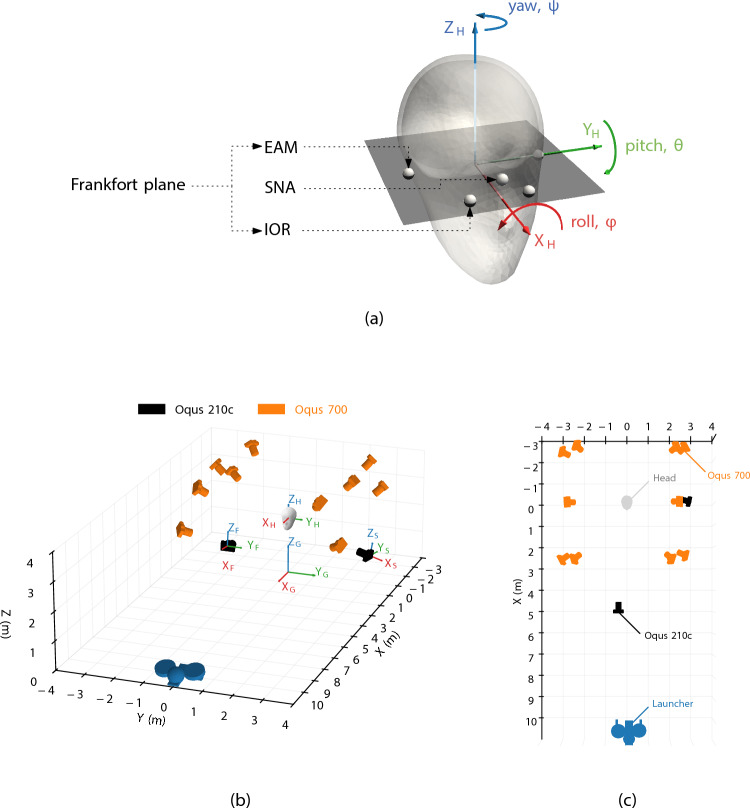
**a** Definition of the marker set consisting of the external auditory meatus (EAM), inferior orbital rim (IOR) and nasion (SNA), used to construct the anatomical coordinate system based on the Frankfort plane. **b** 3D schematic of the experimental setup with reference coordinate systems, where the orange cameras denote the infrared motion capture system (Qualisys Oqus 700+) and the black cameras denote the RGB video cameras (Qualisys Oqus 210c). **c** Top-down view with key components labelled

Ten participants (five male, five female) wore a head-mounted passive marker set (12.5 mm diameter) defining an anatomical coordinate system (ACS) consistent with the Frankfort plane. This plane passes through the lowest point of the eye socket (inferior orbital rim, IOR) and the upper margin of the external auditory meatus (EAM), representing the horizontal axis of the human head [40]. The ACS origin was the midpoint between external auditory meatus (EAM) markers, with the *y*-axis pointing left, the *z*-axis perpendicular to the Frankfort plane (superior), and the *x*-axis anterior along the plane, using markers placed on each inferior orbital rim (IOR) as the reference direction. This ACS has previously been adopted in HAE research [41, 42] and similar football heading protocols [43, 44]. An additional nasion (Skull-NAsion, or SNA) marker, placed according to standard skeletal landmark definitions provided redundancy under occlusion [45]. Figure 1 illustrates the marker configuration and camera layout.

Marker trajectories were captured at 1000 Hz with ten Qualisys Oqus 700+ infrared cameras over a $\approx 4 \times 2 \times 3$ m volume. Two synchronised RGB cameras (Qualisys Oqus 210c) recorded at 50 Hz and 1280 $\times$ 720 pixels with a 1/250 s per frame shutter speed, providing frontal (F) and side (S) views located at (5.0, –0.4, 2.6) m and (–0.1, 3.0, 0.9) m in the global frame.

The protocol complied with Football Association heading guidance [46], with each participant performing ten headers. Balls were launched from 10 m at $\approx 20$ m/s (adjusted for ability) using a motorised, twin-wheel JUGS soccer ball launcher (JUGS Sports) to ensure consistent delivery. Participants directed five headers back toward the launcher (linear, L) and five toward a target 90$^\circ$ to the right (rotational, R). Trials were retained if head–ball contact occurred.

**Fig. 2:**
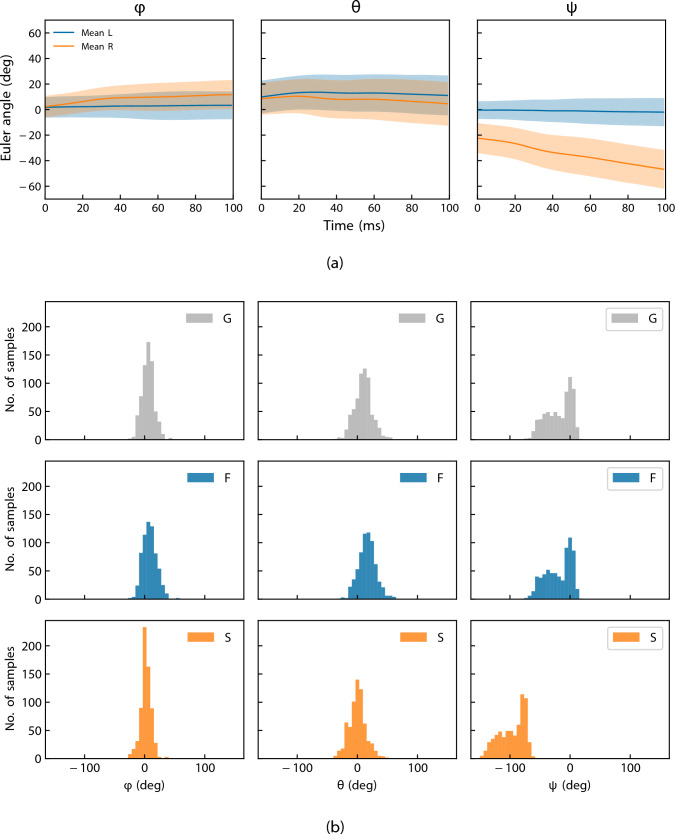
Summary of reference pose measurements obtained from motion capture: **a** Mean and standard deviation of head pose Euler angles for linear (L) and rotational (R) headers. **b** Distribution of ground-truth head pose angles across coordinate systems: global (G), frontal camera (F), and side camera (S)

For each trial, a 100 ms window (20 ms pre- and 80 ms post-impact) was extracted, consistent with recommended HAE windows [47, 48], yielding six RGB frames and 100 motion-capture frames per header. Marker trajectories were low-pass filtered with a fourth-order, zero-lag Butterworth filter; the cut-off (50 Hz) was chosen following residual analysis [13, 49] and frequency-domain inspection (fast Fourier transform magnitudes checked against a 10% threshold [12]). Rigid body head orientation was reconstructed in Qualisys Track Manager using a 5 mm bone tolerance and 10 mm residual to reduce skin artefacts, and visually verified against the synchronised video to ensure tracking fidelity. All resulting orientations represent the rotation of the head relative to the global coordinate system, and therefore encapsulate both neck flexion/torsion and whole-body rotation during the heading motion. Figure 2 summarises the resulting Euler angle trajectories and pose distributions, highlighting the larger yaw angles observed by the side camera.

### Models and inputs

The selected deep learning models represent complementary approaches. 6DRepNet360 [39] is a lightweight convolutional neural network that directly regresses head pose using a 360$^\circ$ formulation for extreme angles. VGGHeads [30] is a multi-task network that performs head detection and 3D face-mesh reconstruction, from which pose is inferred. SAM 3D Body [35] is a full-body human mesh recovery model that uses a promptable encoder–decoder and the Momentum Human Rig [32] to estimate coherent whole-body pose and shape, including global head orientation. Inputs followed each model’s expected format: VGGHeads on uncropped frames, 6DRepNet360 on loosely cropped head images, and SAM 3D on full-body crops.

To ensure standardised inputs, detections were obtained prior to pose estimation. Full-body detections for SAM 3D were produced by a ViTDet-based detector [50], which successfully identified the participants in all frames. Head detection was more challenging because the face was frequently occluded by the ball, hands, or arms. Therefore, a small benchmarking experiment compared the performance of three detectors: RetinaFace, YOLOv8 trained on SCUT-HEAD [51, 52], and VGGHeads’ native head detector. Ground-truth head boxes were manually annotated and when the head was largely occluded, box positions were estimated using adjacent frames. Following Zhou et al. [53], a 50% context margin was added to all crops.

Detection accuracy was then measured using intersection over union [54], with a lenient success threshold of an intersection over union of 0.3 to account for imperfect alignment between face and head crops. The best-performing head detections were then used as input to 6DRepNet360.

### Postprocessing

#### Anatomical alignment

Pose estimation models typically express head orientation relative to the camera coordinate system (C). To compare these predictions with the reference motion-capture orientations, camera-relative predictions of head rotation, $\hat{R}_{\textrm{HC}}$ were converted to predicted rotation of the head in the global frame, $\hat{R}_{\textrm{HG}}$, using the calibrated extrinsic parameters of each camera relative to the global frame, $R_{\textrm{GC}}$, as follows:

1 $$\begin{aligned} \hat{R}_{\textrm{HG}} = R_{\textrm{GC}} \, \hat{R}_{\textrm{HC}} . \end{aligned}$$

However, systematic discrepancies can still arise due to minor differences in anatomical coordinate system (ACS) definitions between deep learning model training datasets and the experimental ACS [55]. A process for removing such systematic offsets has been outlined by Cobo et al. [56], in which a single alignment rotation $\Delta$ is estimated and applied to the predicted rotation matrices. For a video sequence with ground-truth orientations $\{R_1, \ldots , R_N\}$ and corresponding predictions $\{\hat{R}_1, \ldots , \hat{R}_N \}$ (both in the global frame), framewise relative errors $\delta _i = \hat{R}_i^{\top } R_i$ were computed, and $\Delta$ was taken as the Karcher mean of $\{\delta _i\}$ on $\textrm{SO}(3)$ using an iterative log–exp averaging procedure [57–59]. Aligned predictions, $\hat{R}_i^{a}$, were then obtained as:

2 $$\begin{aligned} \hat{R}_i^{a} = \hat{R}_i \, \hat{\Delta }^{\top }, \quad i = 1,\dots ,N, \end{aligned}$$

ensuring that predictions and reference orientations share a common ACS.

#### Temporal filtering

All three models are trained for per-frame pose estimation, meaning that temporal sequences of raw predictions obtained from video can exhibit artefacts that are inconsistent with expected head dynamics. A simple temporal smoothing procedure that respects rotation geometry was therefore applied. Rotations were mapped to the Lie algebra $\mathfrak {so}(3)$ via the matrix logarithm. A Gaussian kernel with standard deviation $\sigma$ (frames) was applied in the tangent space, and filtered rotations were mapped back to $\textrm{SO}(3)$ using the matrix exponential, following the approach detailed by Lee and Shin [60]. For each model, the value of $\sigma$ that minimised the chosen evaluation metrics (outlined in detail in Sect. 2.4) was used.

### Evaluation metrics

#### Detection

A head detection was considered correct if the intersection over union between the predicted and ground-truth bounding boxes exceeded a threshold of 0.3 (see Sect. 2.2). Then, for a given set of detections:

3 $$\begin{aligned} \text {Precision}&= \frac{\text {TP}}{\text {TP} + \text {FP}}, \end{aligned}$$

4 $$\begin{aligned} \text {Recall}&= \frac{\text {TP}}{\text {TP} + \text {FN}}, \end{aligned}$$

where $\textrm{TP}$ denotes true positives, $\textrm{FP}$ false positives, and $\textrm{FN}$ false negatives. The F1 score is then used to provide a balanced measure of overall detection performance:

5 $$\begin{aligned} \text {F1} = 2 \times \frac{\text {Precision} \times \text {Recall}}{\text {Precision} + \text {Recall}}. \end{aligned}$$

For all head detection models the precision, recall, F1 score and mean inference time are reported.

#### Pose

The mean absolute error of Euler angles is commonly used in head pose estimation [61] but is sensitive to angle conventions, gimbal lock, and rotation ordering [28, 53]. Therefore, performance was instead assessed using geodesic distance on $\textrm{SO}(3)$, which provides a coordinate-free measure of rotational difference [56, 62]. For ground-truth and predicted rotations *R* and $\hat{R}$, the geodesic error (GE) is defined as the magnitude of the rotation required to align them:

6 $$\begin{aligned} \Delta R = R^\top \hat{R}, \qquad \text {GE} = d(R,\hat{R}) = \cos ^{-1} \left( \frac{\textrm{tr}(\Delta R) - 1}{2} \right) , \end{aligned}$$

where $\textrm{tr}(\cdot )$ denotes the trace operator. The resulting scalar value lies in $[0, \pi ]$ rad, representing the minimal angular displacement between *R* and $\hat{R}$.

To assess temporal consistency, GE is here extended to incremental geodesic error (IGE), to compare the change in orientation between successive frames in the ground-truth and predicted sequences. The relative rotation between consecutive frames in each sequence is first computed as:

7 $$\begin{aligned} \Delta R_{\text {prev}} = R_{\text {prev}}^{\top } R, \qquad \Delta \hat{R}_{\text {prev}} = \hat{R}_{\text {prev}}^{\top } \hat{R}. \end{aligned}$$

The corresponding geodesic distances $d(\Delta R_{\text {prev}})$ and $d(\Delta \hat{R}_{\text {prev}})$ quantify the angular displacement between frames. The incremental geodesic error at each time step is then given by:

8 $$\begin{aligned} \text {IGE} = \left| \, d(\Delta R_{\text {prev}}) - d(\Delta \hat{R}_{\text {prev}}) \, \right| . \end{aligned}$$

which isolates differences in frame-to-frame rotational change. For each model, GE and IGE were computed per frame and summarised using mean and median values.

#### Facial visibility and temporal phase

Facial occlusion arises from the head turning away from the camera and from occluding objects such as the ball or arms. To quantify its effect on prediction accuracy, a visibility ratio was computed per frame. A face bounding box and any occluding objects were manually annotated, and visible face area was defined as the non-overlapping region of the face box. For each camera, area was normalised by the maximum observed value, yielding a visibility ratio in [0, 1]. Because incremental errors are defined between successive frames, the first visibility sample of each sequence was excluded from incremental analyses.

**Fig. 3:**
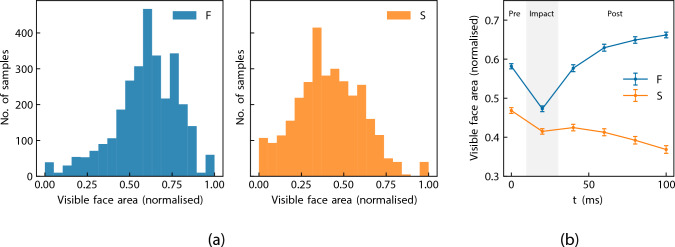
Distributions and temporal dynamics of face visibility ratios across the dataset for cameras F (front) and S (side). **a** Overall distributions of visible face area. **b** Variation in visible face area across the pre-impact, impact, and post-impact temporal phases. All values are normalised relative to the maximum observed facial area (in pixels) for each camera view

To further evaluate estimation accuracy during critical phases of the heading motion, the 100 ms extraction window (comprising six RGB frames at 50 Hz) was also analysed as a continuous time series. Mean visibility, GE, and IGE were computed at each 20 ms time step. The sequence was divided into three conceptual phases: pre-impact ($t=0$ ms), impact ($t=20$ ms, coinciding with closest frame to ball contact), and post-impact ($t=40$ to 100 ms). This time-series analysis (temporal variations illustrated in Fig. 3b) was used to examine how GE and IGE varied alongside dynamic occlusion and rapid kinematic changes over the course of the HAE. This ratio (distribution and temporal variations illustrated in Fig. 3) was used to examine how GE and IGE varied with occlusion severity.

### Implementation details

All experiments were run on a single NVIDIA GeForce RTX 4080 GPU. All rotation operations, including camera-frame transformations, temporal filtering, and error computations, were implemented using rotation matrices to avoid ambiguities associated with quaternion conventions or Euler angle ordering. Euler angles are used only for visualisation to provide interpretable summaries of rotational behaviour throughout. Inference times for each model are reported as mean per-frame values computed over all evaluated frames. Statistical analysis and figure generation were carried out in Python 3.11, with statistical significance assessed at $\alpha = 0.05$. Because error distributions deviated from normality (Shapiro–Wilk, $p<0.001$), non-parametric tests were used in all cases. Paired comparisons between camera views (F vs S) were performed using the Wilcoxon signed-rank test, and unpaired comparisons between trial types (L vs R) were assessed using the Mann–Whitney U test.

## Results

### Head detection

All head detectors achieved high accuracy (Table 1). VGGHeads attained the highest precision (0.9940), recall (0.9896), and F1 score (0.9918), while YOLOv8 achieved comparable performance (F1 = 0.9685) with the fastest inference (0.026 s/frame). RetinaFace showed lower recall (0.8646), indicating greater sensitivity to occlusion.

**Table 1:** Performance of selected head detection approaches on the dataset

Model	Precision	Recall	F1 score	Mean inference time (s)
RetinaFace	0.94	0.86	0.90	0.033
YOLOv8	0.98	0.96	0.97	**0**.**026**
VGGHeads	**0**.**99**	**0**.**99**	**0**.**99**	1.498*

*Inference time includes full face mesh reconstruction and head pose estimation

Bold values indicate the best performance for each metric (highest for precision, recall, and F1 score; lowest for inference time)

Although VGGHeads was the slowest (1.50 s/frame), its inference includes full mesh reconstruction and pose estimation. As inference speed was not a priority, VGGHeads detections were used to maximise data consistency across trials.

### Head pose estimation

#### Filter sensitivity

**Fig. 4:**
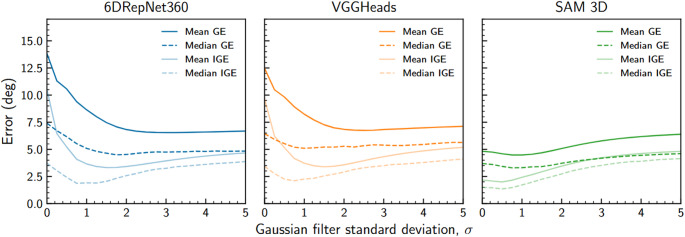
Sensitivity of model errors (both geodesic and incremental geodesic) to standard deviation of Gaussian filtering

Gaussian smoothing improved temporal consistency for all models (Fig. 4). For 6DRepNet360 and VGGHeads, moderate smoothing ($\sigma \approx 1$–2 frames) reduced both GE and IGE, with larger values ($\sigma >3$) attenuating genuine motion. A filter width of $\sigma = 1.5$ was therefore selected for these models. In contrast, SAM 3D exhibited lower baseline noise and achieved optimal performance with a smaller filter width of $\sigma = 0.5$, beyond which further smoothing degraded incremental accuracy. These settings were used for all subsequent analyses.

#### Overall performance

The overall head pose estimation performance is summarised in Table 2. SAM 3D achieved the lowest mean and median GE (4.59$^\circ$ and 3.43$^\circ$) and IGE (1.99$^\circ$ and 1.35$^\circ$), outperforming the head-only models and indicating superior accuracy in both static orientation estimation and short-timescale motion tracking. 6DRepNet360 and VGGHeads performed similarly to one another, with mean GE values of 7.46$^\circ$ and 7.26$^\circ$, respectively, and median GE values around 4.5$^\circ$. Incremental errors followed the same pattern. As expected, 6DRepNet360 provided the fastest inference (0.0049 s/frame) due to its lightweight architecture which directly regresses head pose from small cropped regions, while VGGHeads incurred higher computational cost due to its integrated detection and face mesh reconstruction.

**Table 2:** Performance of selected head pose estimation approaches on the dataset

Model	GE (deg)		IGE (deg)		Mean Inference Time (s)
	Mean	Median	Mean	Median	
6DRepNet360	7.46	4.64	3.32	2.05	**0**.**005**
VGGHeads	7.26	4.50	3.39	2.24	1.453*
SAM 3D	**4**.**59**	**3**.**43**	**1**.**99**	**1**.**35**	0.231

*Inference time includes head detection and full face mesh reconstruction

Bold values indicate the best performance for each metric (lowest error and fastest inference time)

**Fig. 5:**
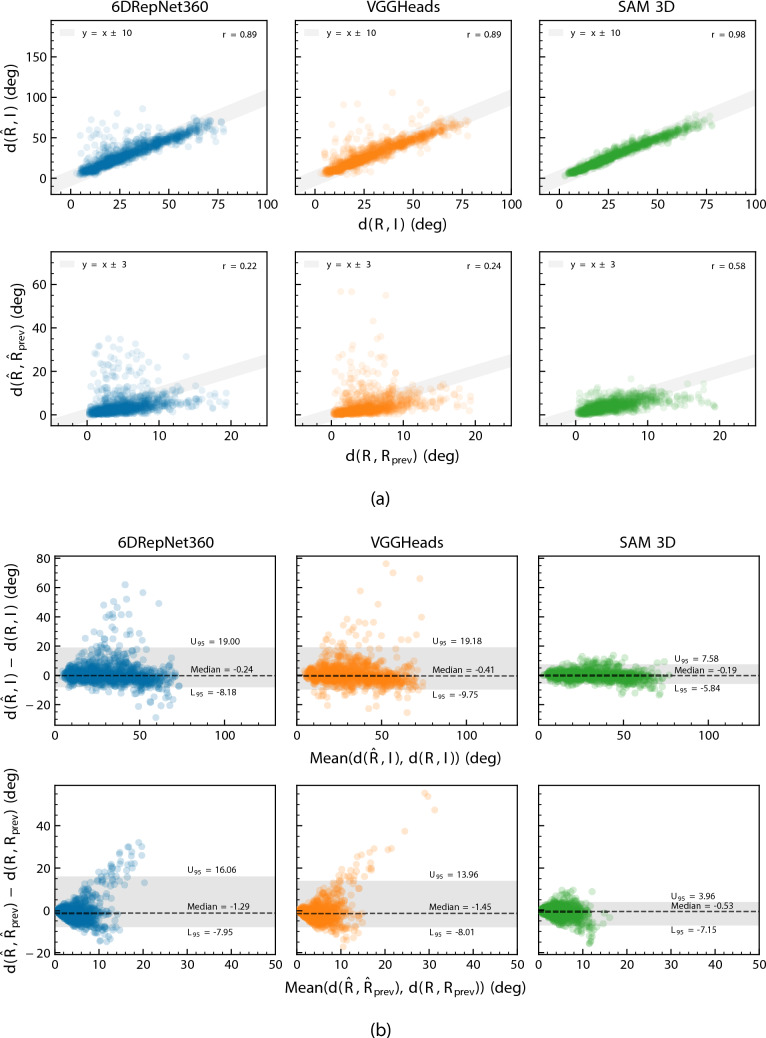
Scatter and Bland–Altman plots of head pose estimation errors. **a** Predicted versus ground-truth head pose angles. **b** Bland–Altman plots showing agreement between predicted and reference orientations

The scatter and Bland–Altman plots in Fig. 5 illustrate frame-wise agreement. Here, *d*(*R*, *I*) denotes the geodesic distance between a rotation *R* and the identity rotation *I*, i.e. the instantaneous magnitude of head rotation, while $d(R, R_{\text {prev}})$ is the geodesic distance between consecutive orientations and thus measures frame-to-frame rotational change. All models showed positive monotonic relationships between predicted and reference values of *d*(*R*, *I*), with Pearson correlation coefficients of $r = 0.89$ for 6DRepNet360 and VGGHeads, and $r = 0.98$ for SAM 3D. Correlations for the incremental measures $d(R, R_{\text {prev}})$ were weaker ($r = 0.22,~0.58$), consistent with the difficulty of capturing high-frequency rotational dynamics from monocular 50 Hz video.

Because normality assumptions were violated (Shapiro–Wilk, $p < 0.001$), Bland–Altman analyses were interpreted using median bias and non-parametric 95% limits of agreement [63]. Across all models and metrics, median biases were small (all $<1.5^\circ$). However, large deviations occurring during extreme head rotations led 6DRepNet360 and VGGHeads to exhibit wide limits of agreement, with upper GE limits around 19$^\circ$ and upper IGE limits of approximately 14$^\circ$ and 16$^\circ$, respectively. SAM 3D, however, showed the smallest dispersion, with limits of agreement falling within $\pm 8^\circ$, reflecting reduced susceptibility to large outliers.

#### Camera and trial type effects

**Fig. 6:**
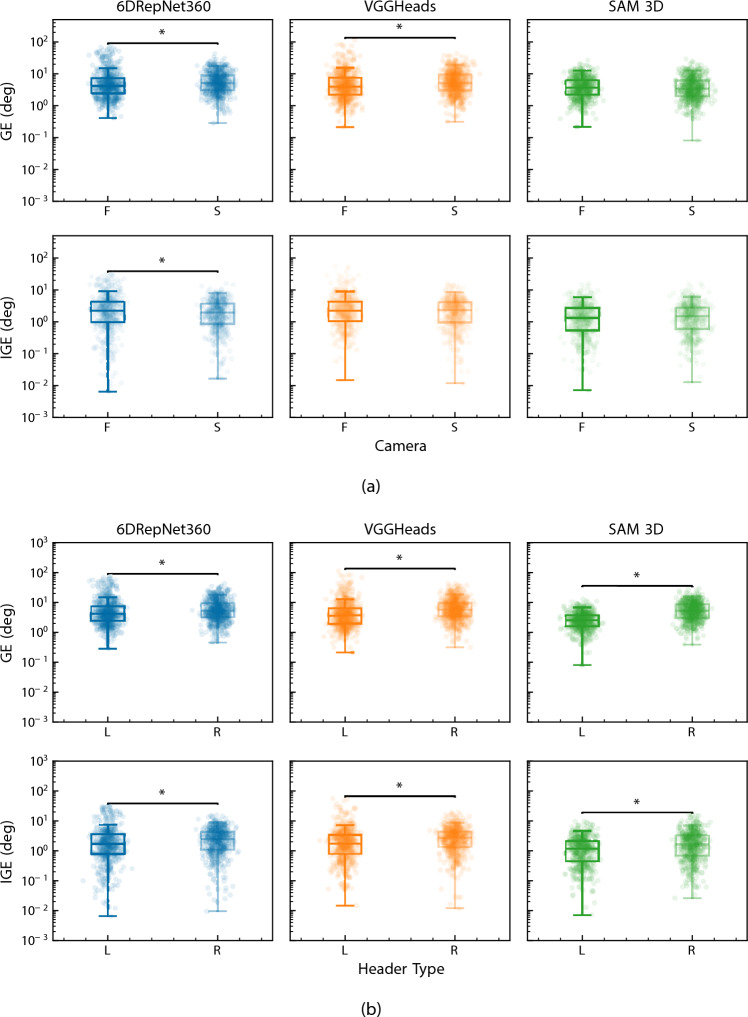
Boxplots of head pose estimation errors. **a** Errors across different camera views. **b** Errors across different trial types. Asterisks indicate statistically significant differences ($p < 0.05$)

The influence of camera view and trial type on pose estimation error (GE and IGE) is summarised in Fig. 6. For paired camera comparisons, effects were observed to be model dependent.

For paired camera comparisons, effects were observed to be model dependent. For GE, both 6DRepNet360 and VGGHeads showed significantly higher errors from the side (S) camera view (6DRepNet360: median increase of 0.90$^\circ$, $p < 0.0001$; VGGHeads: 1.21$^\circ$, effect size $=0.30$, $p < 0.0001$). SAM 3D showed no detectable difference between views (median change of –0.01$^\circ$, $p = 0.64$). Differences were minimal for IGE. 6DRepNet360 showed a statistically significant reduction for the side view (median change of –0.07$^\circ$, $p = 0.023$). VGGHeads showed no significant difference (median change of 0.02$^\circ$, $p = 0.23$). SAM 3D showed a non-significant tendency toward higher IGE in the side view (0.17$^\circ$, $p = 0.057$). Overall, side-view frames produced modest increases in GE for 6DRepNet360 and VGGHeads, while SAM 3D was largely insensitive to viewpoint.

Trial-type comparisons revealed larger differences. Across all models, errors were significantly higher for rotational (R) than linear (L) trials (Mann–Whitney U, all $p < 0.0001$). For GE, median increases were 1.14$^\circ$ for 6DRepNet360, 2.13$^\circ$ for VGGHeads, and 2.84$^\circ$ for SAM 3D. Corresponding effect sizes indicated small-to-moderate differences (Cliff’s $\delta = -0.16$, $-0.28$, and $-0.56$, respectively). The same pattern was observed for IGE, with median increases of 0.84$^\circ$ (6DRepNet360), 1.04$^\circ$ (VGGHeads), and 0.45$^\circ$ (SAM 3D), and Cliff’s $\delta$ values ranging between $-0.21$ and $-0.17$. These findings confirm that large, multi-axis head rotations in R trials introduce greater error across all models (most notably for SAM 3D in terms of GE) reflecting the increased challenge posed by the rapid 3D rotation, self-occlusion, and off-axis motion that the rotational heading action promotes.

#### Face visibility and temporal phase effects

**Fig. 7:**
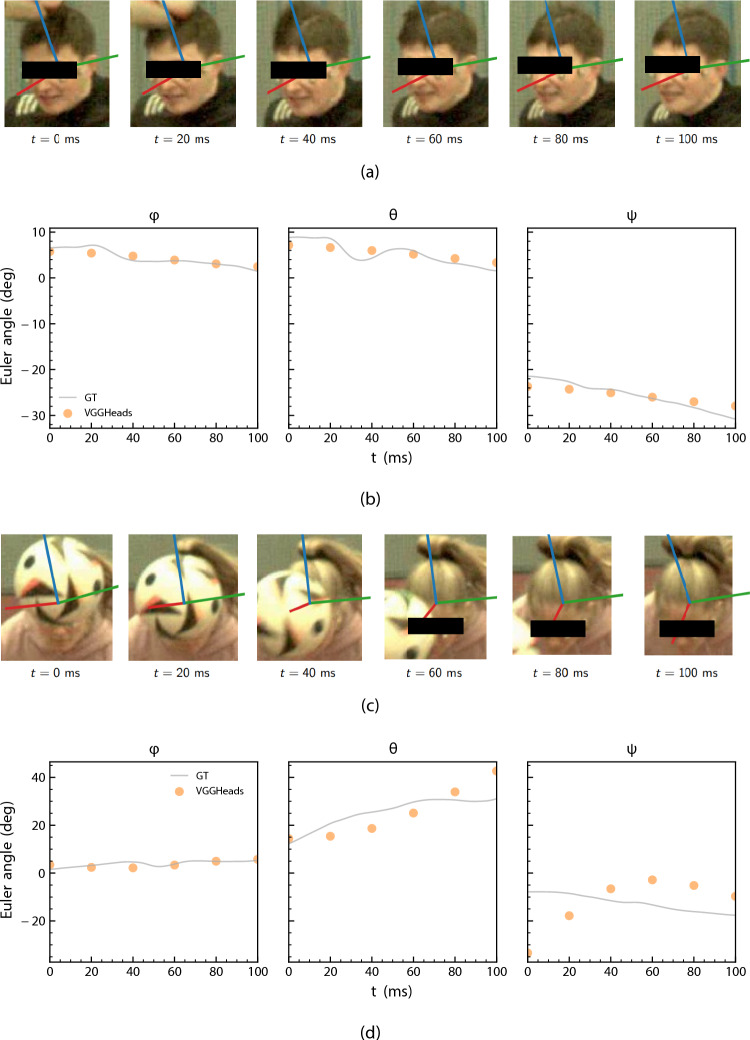
Examples of well-performing (**a**, **b**) and poorly-performing (**c**, **d**) sequences for VGGHeads. Blue, red, and green axes denote the global *x*, *y*, and *z* directions, respectively

**Fig. 8:**
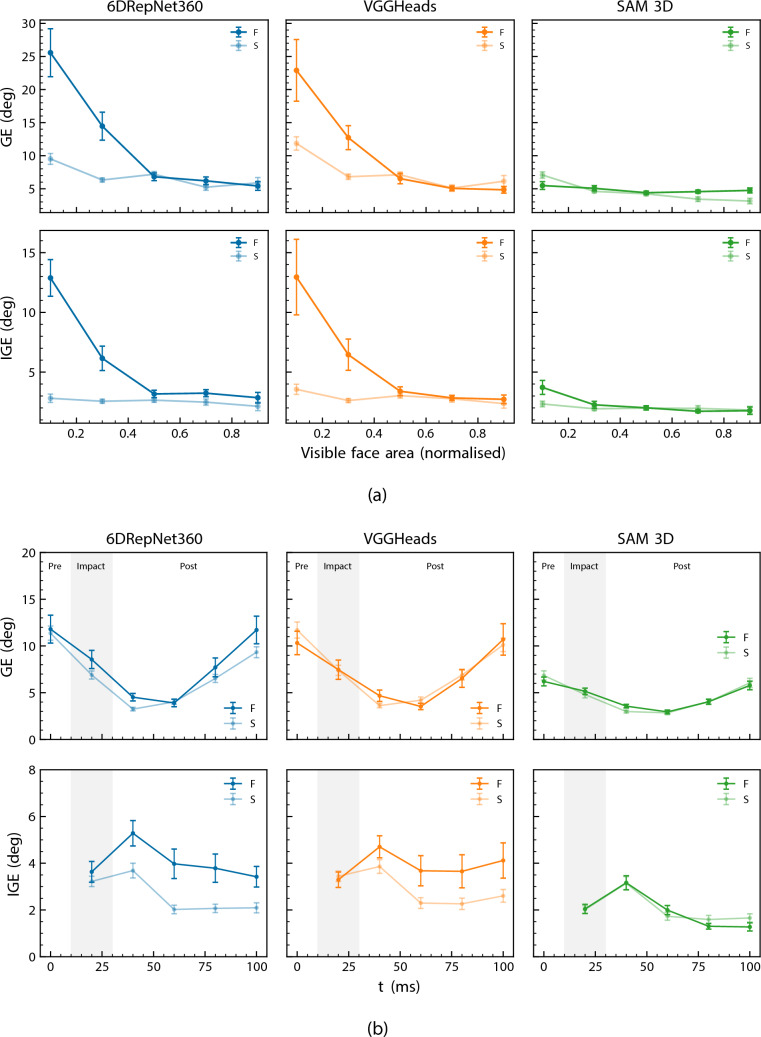
Relationship between head pose estimation error, face visibility ratio, and time. Mean geodesic and incremental geodesic errors across **a** visible face area bins and **b** time, with pre-impact, impact and post-impact phases labelled. Error bars denote standard error

Representative examples of well- and poorly-performing sequences for VGGHeads are shown in Fig. 7. In well-performing examples (Figs. 7a, b), predicted orientations closely followed ground-truth trajectories when the face remained largely visible. In contrast, heavily occluded sequences (Figs. 7c, d) resulted in underestimation of large pitch and yaw angles, producing visibly inaccurate trajectories. These occlusion-driven errors are quantified across the broader dataset in Fig. 8.

Figure 8a quantifies this effect by relating head pose estimation error to the normalised visible face area. For both 6DRepNet360 and VGGHeads, GE and IGE decreased as visibility increased, particularly for frontal-camera views where the main source of occlusion was the ball. VGGHeads exhibited the greatest dependence: mean GE exceeded 20$^\circ$ for frames with $<0.2$ visibility but fell below 5$^\circ$ once the visible area exceeded 0.8. 6DRepNet360 followed the same pattern, though with lower sensitivity at moderate occlusion levels. SAM 3D displayed weaker dependence on face visibility; mean errors remained low (GE <8$^\circ$, IGE <4$^\circ$) even under heavy occlusion, and the shallow slope across visibility bins indicates that its predictions rely less on explicit facial appearance. Furthermore, while the face-dependent models exhibited statistically significant differences between frontal and side camera views, SAM 3D error rates remained consistent ($p = 0.64$) across both viewpoints.

Temporal error dynamics are shown in Fig. 8b. GE followed a U-shaped profile across all models: errors were elevated at the sequence boundaries ($t=0$ and $t=100$ ms) and lowest in the middle of the extraction window ($t=40$–60 ms). This pattern was consistent across camera views. IGE exhibited a different temporal structure: all models showed peak IGE at $t=40$ ms, immediately following the impact phase, with lower values at the sequence boundaries. This IGE peak coincided with the recovery from ball occlusion visible in Fig. 3b.

## Discussion

### Model performance

Across all models, mean geodesic errors (GE) remained within 4–8$^\circ$ and incremental geodesic errors (IGE) below 4$^\circ$, indicating that deep learning methods can recover head orientation from monocular video under the dynamic motion and occlusion conditions typical of sports impacts. Among the evaluated models, SAM 3D produced the lowest mean and median GE and IGE, while also exhibiting the smallest dispersion of errors and the tightest limits of agreement. Its performance maintained GE below 8$^\circ$ and IGE below 4$^\circ$ across visibility levels, reflecting robustness across low-visibility conditions. By jointly estimating body configuration and head orientation using the Momentum Human Rig and a promptable encoder–decoder architecture, SAM 3D can estimate head pose even when facial appearance cues are weak or absent. This contrasts with the explicitly head-driven models, which degrade with reduced visibility.

VGGHeads also yielded lower errors, particularly when the face was clearly visible, benefiting from its dense 3D morphable model reconstruction. However, its per-frame inference cost remains high (over 1 s/frame), limiting practicality for large-scale or real-time applications. 6DRepNet360 delivered similar accuracy with lower inference time. Its lightweight backbone and 360$^\circ$ formulation provided accurate estimates for large yaw rotations, consistent with expectations from its training data. However, its reliance on cropped head images makes it more susceptible to occlusion due to a lack of additional contextual clues, contributing to larger errors in low-visibility conditions.

SAM 3D outperformed previous state of the art human mesh recovery models from recent years. Hybrik-X [64] was benchmarked on the same dataset and showed higher errors (mean GE $=8.06^\circ$; mean IGE $=3.97^\circ$). This direct comparison highlights the rapid rate at which human-mesh-recovery methods have advanced, with SAM 3D improving accuracy by almost a factor of two in some metrics while also producing more stable and anatomically coherent estimates across frames. Such progress suggests that future iterations of dedicated head pose models may increasingly supersede the approaches considered here.

### Viewpoint, motion and occlusion effects

All error distributions were non-Gaussian, so non-parametric tests were used throughout. Differences between front (F) and side (S) camera views were small in magnitude but statistically detectable for most models. For 6DRepNet360 and VGGHeads, geodesic error (GE) was consistently higher for side views (median differences of 0.9°–1.2°). This is consistent with the increased yaw angles and reduced facial visibility characteristic of profile views. In contrast, SAM 3D showed no meaningful GE difference between views (median difference $<0.1^\circ$). For incremental geodesic error (IGE), directional effects were weak or inconsistent across models, indicating that short-term temporal smoothness is largely unaffected by view.

Trial type had a clearer influence. Across all models, rotational (R) trials produced higher errors than linear (L) trials for both GE and IGE. Median differences ranged from 1.1° to 2.8° for GE and 0.45° to 1.36° for IGE, corresponding with small-to-moderate effect sizes. This reflects the greater difficulty of estimating head pose during large, multi-axis rotations, where motion blur, out-of-plane movement, and self-occlusion are more prominent. In particular, these behaviours are rare in standard pose estimation datasets, demonstrating how errors can arise due to distributional mismatch between the sports head impacts considered here and the typical training data of the deep learning models.

Face visibility influenced error for the dedicated head pose models, but had only a minor effect on SAM 3D (Fig. 8). For 6DRepNet360 and VGGHeads, GE exceeded 20$^\circ$ when visibility fell below 20%, decreasing to below 5$^\circ$ when most of the face was visible. In contrast, SAM 3D maintained low errors across all visibility bins, with only marginal improvement as visible area increased. This aligns with its full-body formulation: by leveraging body configuration, SAM 3D can produce accurate head pose estimates even when facial appearance cues are heavily degraded or occluded. Qualitative examples further illustrated these trends, with 6DRepNet360 and VGGHeads seen to degrade during ball impact, arm occlusions, or out-of-plane motion, while SAM 3D remained comparatively stable.

The U-shaped GE profile seen across the temporal window (Fig. 8b) is a result of the anatomical alignment procedure. Head orientation changes continuously during the heading motion, ranging from approximately 22° to 36° from neutral. The global alignment (see Sect. 2.3) calculates a single best fit for the entire sequence, which naturally centres on the middle frames and pushes residual error out to the boundaries. Therefore, the lower GE values in the middle frames (such as the sub-4° errors for SAM 3D between $t=40$ and 60 ms) provide a more accurate reflection of true model performance. This alignment step is still essential to correct the systematic differences between definitions of the model and experimental setup coordinate systems. Without it, mean errors can artificially inflate to over 20° simply due to a coordinate mismatch.

Unlike GE, IGE measures frame-to-frame consistency and is unaffected by the global alignment step. This makes it a clearer indicator of dynamic tracking vulnerabilities over time. The peak in IGE at $t=40$ ms highlights a tracking lag immediately following the impact phase. Because IGE quantifies the rotational change between consecutive frames, the elevated error at this point captures the difficult transition from the heavily occluded impact frame at $t=20$ ms to the clearer post-impact frame, compounded by rapid angular acceleration. This tracking failure was particularly obvious for the frontal camera, where the ball caused an abrupt drop in visibility. While SAM 3D also exhibited an increase in IGE at this time step, it maintained a lower overall error magnitude. This aligns with its ability to leverage visible body context to constrain the pose estimate even when features of the head are temporarily occluded.

### Limitations

The dataset used in this study provides a controlled, high-fidelity environment for benchmarking pose estimators during football headers, however certain limitations must be acknowledged. Its controlled laboratory setting, while enabling precise ground-truth motion capture, does not capture the complete environmental variability, background complexity, or multi-player occlusion typical of real gameplay. Nonetheless, the dataset represents a valuable first step toward sports-specific benchmarking and is more challenging than established head pose datasets such as BIWI [37] or CMU-Panoptic [38], owing to its faster motion, larger rotations, and frequent occlusion.

A further limitation is dataset scale (1.2k images across 10 participants), which restricts opportunities for fine-tuning or model adaptation and reflects the broader difficulty of collecting large HAE datasets with high quality annotations. The 50 Hz sampling rate of the RGB cameras (typical of consumer and broadcast imaging) also prevents accurate derivation of higher-order kinematics such as angular velocity or acceleration. As a result, this study evaluates pose estimation accuracy at the level of orientation only, and does not assess the suitability of current models for estimating full HAE kinematics or supporting downstream biomechanical simulation pipelines, where small rotational errors are amplified through numerical differentiation.

### Practical implications and future research

Despite these limitations, the results indicate potential for incorporating modern deep learning models into semi-automated HAE reconstruction workflows. SAM 3D, in particular, achieved the highest accuracy and was robust to occlusion and viewpoint changes, making it the most promising candidate for practical deployment. Lighter models focused solely on the head pose estimation task such as 6DRepNet360 may still have relevance due to their near real-time efficiency, but SAM 3D provides the most reliable performance across the full range of sports-relevant conditions encountered here. A practical next step could be to use models such as SAM 3D to produce reliable initial pose estimates that are subsequently refined by human raters, reducing the manual labour typically associated with model-based image matching (MBIM) [26, 65].

Several additional research directions could further increase the reliability and applicability of such models in sports HAE environments. Pre- and post-processing methods including: motion deblurring [65], frame interpolation [66], and learned temporal upsampling or interpolation [67, 68] may help to mitigate temporal sparsity and motion blur inherent in standard video frame rates. More fundamentally, the development of large-scale, in-the-wild HAE pose datasets, analogous to initiatives such as WorldPose [69] but specific to HAEs, will be essential for training and improving models that generalise beyond laboratory conditions.

Furthermore, while this study evaluated unhelmeted impacts, these findings have important implications for helmeted sports, such as American football, ice hockey or cricket. The additional facial occlusion caused by helmets and visors would likely render explicitly head-dependent models (e.g., 6DRepNet360, VGGHeads) ineffective without further fine-tuning on sport-specific datasets. In contrast, full-body HMR models like SAM 3D, which infer head orientation from the broader kinematic context of the visible torso and limbs, are theoretically better equipped to handle helmeted scenarios, though verifying this remains an important area for future validation.

Ultimately, the ability to derive reliable head pose estimates directly from video offers a scalable, non-invasive pathway for quantifying HAEs. Progressively evaluating models under increasingly realistic conditions, including broadcast or match-play footage, and integrating data-driven predictions with computational biomechanical simulations, may enable new hybrid video–simulation frameworks for sports injury research and prevention.

## Conclusions

This study benchmarked three deep learning models for monocular head pose estimation during football headers. All models recovered head orientation with single-digit geodesic errors, but the full-body human mesh recovery model (SAM 3D) delivered the highest accuracy and robustness, outperforming explicitly face-dependent models (6DRepNet360, VGGHeads). Time-resolved analysis revealed that while static orientation accuracy is primarily limited by large out-of-plane rotations, dynamic tracking is most vulnerable immediately following ball contact due to transient occlusion. Face-dependent models destabilised during this phase, whereas SAM 3D remained stable. These findings indicate that modern full-body models can recover head orientation with promising accuracy under challenging sports conditions, offering a near-term practical solution for semi-automating model-based image matching (MBIM) workflows. Ultimately, fielding fully automated, non-invasive methods for analysing head acceleration events will require large-scale, in-the-wild datasets and models robust to heavy occlusion.

## Data Availability

The datasets generated and analysed during this study are not publicly available due to ethical and privacy constraints associated with human participant data, but can be requested by contacting the authors of this article, subject to institutional approval.

## References

[1] Tierney G (2024) Concussion biomechanics, head acceleration exposure and brain injury criteria in sport: A review. Sports Biomechanics 23(11):1888–1916. 10.1080/14763141.2021.2016929 34939531 10.1080/14763141.2021.2016929

[2] Tooby J, Woodward J, Tucker R, Jones B, Falvey É, Salmon D, Bussey MD, Starling L, Tierney G (2024) Instrumented Mouthguards in Elite-Level Men’s and Women’s Rugby Union: The Incidence and Propensity of Head Acceleration Events in Matches. Sports Med 54(5):1327–1338. 10.1007/s40279-023-01953-7 37906425 10.1007/s40279-023-01953-7 PMC11127838

[3] Daneshvar DH, Nowinski CJ, McKee A, Cantu RC (2011) The Epidemiology of Sport-Related Concussion. Clin Sports Med 30(1):1–17. 10.1016/j.csm.2010.08.006 21074078 10.1016/j.csm.2010.08.006 PMC2987636

[4] Daniel RW, Rowson S, Duma SM (2012) Head Impact Exposure in Youth Football. Ann Biomed Eng 40(4):976–981. 10.1007/s10439-012-0530-7 22350665 10.1007/s10439-012-0530-7 PMC3310979

[5] Mackay DF, Russell ER, Stewart K, MacLean JA, Pell JP, Stewart W (2019) Neurodegenerative Disease Mortality among Former Professional Soccer Players. N Engl J Med 381(19):1801–1808. 10.1056/NEJMoa1908483 31633894 10.1056/NEJMoa1908483 PMC8747032

[6] McKee AC, Cantu RC, Nowinski CJ, Hedley-Whyte ET, Gavett BE, Budson AE, Santini VE, Lee H-S, Kubilus CA, Stern RA (2009) Chronic traumatic encephalopathy in athletes: Progressive tauopathy after repetitive head injury. J Neuropathol Exp Neurol 68(7):709–735. 10.1097/NEN.0b013e3181a9d503 19535999 10.1097/NEN.0b013e3181a9d503 PMC2945234

[7] Russell ER, Mackay DF, Stewart K, MacLean JA, Pell JP, Stewart W (2021) Association of Field Position and Career Length With Risk of Neurodegenerative Disease in Male Former Professional Soccer Players. JAMA Neurol 78(9):1057–1063. 10.1001/jamaneurol.2021.2403 34338724 10.1001/jamaneurol.2021.2403 PMC8329793

[8] Russell ER, Mackay DF, Lyall D, Stewart K, MacLean JA, Robson J, Pell JP, Stewart W (2022) Neurodegenerative disease risk among former international rugby union players. Journal of Neurology Neurosurgery & Psychiatry 93(12):1262–1268. 10.1136/jnnp-2022-329675. Chap. Neurodegeneration 10.1136/jnnp-2022-329675 PMC9669247 36195436

[9] Stewart W, Buckland ME, Abdolmohammadi B, Affleck AJ, Alvarez VE, Gilchrist S, Huber BR, Lee EB, Lyall DM, Nowinski CJ, Russell ER, Stein TD, Suter CM, McKee AC (2023) Risk of chronic traumatic encephalopathy in rugby union is associated with length of playing career. Acta Neuropathol 146(6):829–832. 10.1007/s00401-023-02644-3 37872234 10.1007/s00401-023-02644-3 PMC10627955

[10] Arbogast KB, Caccese JB, Buckley TA, McIntosh AS, Henderson K, Stemper BD, Solomon G, Broglio SP, Funk JR, Crandall JR (2022) Consensus Head Acceleration Measurement Practices (CHAMP): Origins, Methods, Transparency and Disclosure. Ann Biomed Eng 50(11):1317–1345. 10.1007/s10439-022-03025-9 35920964 10.1007/s10439-022-03025-9 PMC9652170

[11] Stark NE-P, Henley ES, Reilly BA, Kuehl DR, Rowson S (2025) Kinematic Insights Into Older Adult Fall-Related Head Impacts: Boundary Conditions and Injury Risk. J Am Med Dir Assoc 26(5):105545. 10.1016/j.jamda.2025.105545 40088941 10.1016/j.jamda.2025.105545

[12] Bailey A, Funk J, Lessley D, Sherwood C, Crandall J, Neale W, Rose N (2018) Validation of a videogrammetry technique for analysing American football helmet kinematics. Sports Biomechanics 19(5):678–700. 10.1080/14763141.2018.1513059 30274537 10.1080/14763141.2018.1513059

[13] Gyemi DL, Jadischke R, Andrews DM (2023) Validation of a multi-camera videogrammetry approach for quantifying helmet impact velocity in football. Sports Eng 26(1):31. 10.1007/s12283-023-00423-7

[14] England R (2025) Advanced biofidelic headforms for high fidelity re-enactment of real-world head impacts in cricket. PhD thesis, Loughborough University. 10.26174/thesis.lboro.28323377.v1

[15] Yuan Q, Li X, Zhou Z, Kleiven S (2024) A novel framework for video-informed reconstructions of sports accidents: A case study correlating brain injury pattern from multimodal neuroimaging with finite element analysis. Brain Multiphysics 6:100085. 10.1016/j.brain.2023.100085

[16] Blythman R, Saxena M, Tierney GJ, Richter C, Smolic A, Simms C (2022) Assessment of deep learning pose estimates for sports collision tracking. J Sports Sci 40(17):1885–1900. 10.1080/02640414.2022.2117474 36093680 10.1080/02640414.2022.2117474

[17] Neale W, Held JS, Jadischke R, Rundell S, Arrington D (2022) Video Analysis of Head Acceleration Events

[18] Funk JR, McIntosh AS, Withnall C, Wonnacott M, Jadischke R (2022) Best Practices for Conducting Physical Reconstructions of Head Impacts in Sport. Ann Biomed Eng 50(11):1409–1422. 10.1007/s10439-022-03024-w 35876938 10.1007/s10439-022-03024-w

[19] Aston T, Teixeira-Dias F (2025) Quantitative video analysis of head acceleration events: A review. Frontiers in Bioengineering and Biotechnology 13. 10.3389/fbioe.2025.1658222 10.3389/fbioe.2025.1658222 PMC12405429 40909217

[20] Gyemi DL, Andrews DM, Jadischke R (2021) Three-dimensional video analysis of helmet-to-ground impacts in North American youth football. J Biomech 125:110587. 10.1016/j.jbiomech.2021.110587 34274559 10.1016/j.jbiomech.2021.110587

[21] Post A, Koncan D, Kendall M, Cournoyer J, Michio Clark J, Kosziwka G, Chen W, de Grau Amezcua S, Blaine Hoshizaki T (2018) Analysis of speed accuracy using video analysis software. Sports Eng 21(3):235–241. 10.1007/s12283-018-0263-4

[22] Krosshaug T, Bahr R (2005) A model-based image-matching technique for three-dimensional reconstruction of human motion from uncalibrated video sequences. J Biomech 38(4):919–929. 10.1016/j.jbiomech.2004.04.033 15713313 10.1016/j.jbiomech.2004.04.033

[23] Bailey AM, Sherwood CP, Funk JR, Crandall JR, Carter N, Hessel D, Beier S, Neale W (2020) Characterization of Concussive Events in Professional American Football Using Videogrammetry. Ann Biomed Eng 48(11):2678–2690. 10.1007/s10439-020-02637-3 33025319 10.1007/s10439-020-02637-3

[24] Jadischke R, Zendler J, Lovis E, Elliott A, Goulet G (2019) Development of a Methodology and Preliminary Analysis of Head Impacts in American 7-v-7 Non-Tackle Football. In: Proceedings of the IRCOBI Conference 2019, Florence, Italy

[25] Jadischke R, Zendler J, Lovis E, Elliott A, Goulet GC (2020) Quantitative and qualitative analysis of head and body impacts in American 7v7 non-tackle football. BMJ Open Sport & Exercise Medicine 6(1). 10.1136/bmjsem-2019-000638 10.1136/bmjsem-2019-000638 PMC7011012 32095268

[26] Tierney GJ, Gildea K, Krosshaug T, Simms CK (2019) Analysis of ball carrier head motion during a rugby union tackle without direct head contact: A case study. International Journal of Sports Science & Coaching 14(2):190–196. 10.1177/1747954119833477

[27] Yamazaki J, Gilgien M, Kleiven S, Mcintosh AS, Nachbauer W, Müller E, Bere T, Bahr R, Krosshaug T (2015) Analysis of a Severe Head Injury in World Cup Alpine Skiing. Medicine & Science in Sports & Exercise 47(6):1113. 10.1249/MSS.0000000000000511 25207934 10.1249/MSS.0000000000000511

[28] Hempel T, Abdelrahman AA, Al-Hamadi A (2022) 6D Rotation Representation For Unconstrained Head Pose Estimation. In: 2022 IEEE International Conference on Image Processing (ICIP), pp. 2496–2500. 10.1109/ICIP46576.2022.9897219

[29] Algabri R, Abdu A, Lee S (2024) Deep learning and machine learning techniques for head pose estimation: A survey. Artif Intell Rev 57(10):288. 10.1007/s10462-024-10936-7

[30] Kupyn O, Khvedchenia E, Rupprecht C (2024) VGGHeads: 3D Multi Head Alignment with a Large-Scale Synthetic Dataset

[31] Loper M, Mahmood N, Romero J, Pons-Moll G, Black MJ (2015) SMPL: A skinned multi-person linear model. ACM Transactions on Graphics 34(6):248–124816. 10.1145/2816795.2818013

[32] ...Ferguson A, Osman AAA, Bescos B, Stoll C, Twigg C, Lassner C, Otte D, Vignola E, Prada F, Bogo F, Santesteban I, Romero J, Zarate J, Lee J, Park J, Yang J, Doublestein J, Venkateshan K, Kitani K, Kavan L, Farra MD, Hu M, Cioffi M, Fabris M, Ranieri M, Modarres M, Kadlecek P, Khirodkar R, Abdrashitov R, Prévost R, Rajbhandari R, Mallet R, Pearsall R, Kao S, Kumar S, Parrish S, Yu S-I, Saito S, Shiratori T, Wang T-L, Tung T, Xu Y, Dong Y, Chen Y, Xu Y, Ye Y, Jiang Z (2025) MHR: Momentum Human Rig. arXiv. 10.48550/arXiv.2511.15586

[33] Li J, Xu C, Chen Z, Bian S, Yang L, Lu C (2021) HybrIK: A Hybrid Analytical-Neural Inverse Kinematics Solution for 3D Human Pose and Shape Estimation. In: Proceedings of the IEEE/CVF International Conference on Computer Vision and Pattern Recognition, Nashville, TN, USA. 10.48550/arXiv.2011.14672

[34] Goel S, Pavlakos G, Rajasegaran J, Kanazawa A, Malik J (2023) Humans in 4D: Reconstructing and Tracking Humans with Transformers. In: Proceedings of the IEEE/CVF International Conference on Computer Vision and Pattern Recognition, Paris, France. 10.48550/arXiv.2305.20091

[35] Yang X, Kukreja D, Pinkus D, Sagar A, Fan T, Park J, Cao J, Liu J, Ugrinovic N, Feiszli M, Malik J, Dollar P, Kitani K (2025) SAM 3D Body: Robust Full-Body Human Mesh Recovery. arXiv

[36] Gildea K, Hall D, Cherry CR, Simms C (2024) Forward dynamics computational modelling of a cyclist fall with the inclusion of protective response using deep learning-based human pose estimation. J Biomech 163:111959. 10.1016/j.jbiomech.2024.111959 38286096 10.1016/j.jbiomech.2024.111959

[37] Fanelli G, Dantone M, Gall J, Fossati A, Van Gool L (2013) Random Forests for Real Time 3D Face Analysis. Int J Comput Vision 101(3):437–458. 10.1007/s11263-012-0549-0

[38] Joo H, Simon T, Li X, Liu H, Tan L, Gui L, Banerjee S, Godisart T, Nabbe B, Matthews I, Kanade T, Nobuhara S, Sheikh Y (2015) Panoptic Studio: A Massively Multiview System for Social Interaction Capture. In: Proceedings of the IEEE International Conference on Computer Vision, Santiago, Chile. 10.48550/arXiv.1612.03153 10.1109/TPAMI.2017.2782743 29990012

[39] Hempel T, Abdelrahman AA, Al-Hamadi A (2024) Toward Robust and Unconstrained Full Range of Rotation Head Pose Estimation. IEEE Trans Image Process 33:2377–2387. 10.1109/TIP.2024.3378180 38512742 10.1109/TIP.2024.3378180

[40] Yoganandan N, Pintar FA, Zhang J, Baisden JL (2009) Physical properties of the human head: Mass, center of gravity and moment of inertia. J Biomech 42(9):1177–1192. 10.1016/j.jbiomech.2009.03.029 19428013 10.1016/j.jbiomech.2009.03.029

[41] Abrams MZ, Venkatraman J, Sherman D, Ortiz-Paparoni M, Bercaw JR, MacDonald RE, Kait J, Dimbath ED, Pang DY, Gray A, Luck JF, Bir CA, Bass CR (2024) Biofidelity and Limitations of Instrumented Mouthguard Systems for Assessment of Rigid Body Head Kinematics. Ann Biomed Eng 52(10):2872–2883. 10.1007/s10439-024-03563-4 38910203 10.1007/s10439-024-03563-4

[42] Campbell KR, Warnica MJ, Levine IC, Brooks JS, Laing AC, Burkhart TA, Dickey JP (2016) Laboratory Evaluation of the gForce Tracker, a Head Impact Kinematic Measuring Device for Use in Football Helmets. Ann Biomed Eng 44(4):1246–1256. 10.1007/s10439-015-1391-7 26198174 10.1007/s10439-015-1391-7

[43] Caccese JB, Buckley TA, Tierney RT, Arbogast KB, Rose WC, Glutting JJ, Kaminski TW (2018) Head and neck size and neck strength predict linear and rotational acceleration during purposeful soccer heading. Sports Biomechanics 17(4):462–476. 10.1080/14763141.2017.1360385 29037111 10.1080/14763141.2017.1360385

[44] Shewchenko N, Withnall C, Keown M, Gittens R, Dvorak J (2005) Heading in football. Part 1: Development of biomechanical methods to investigate head response. British Journal of Sports Medicine 39 Suppl 1(Suppl 1), 10–25. 10.1136/bjsm.2005.019034 10.1136/bjsm.2005.019034 PMC1765311 16046351

[45] van Sint Jan S (2007) Color Atlas of Skeletal Landmark Definitions, 1st edn. Elsevier Health Sciences, St. Louis

[46] Heading in Football. https://www.englandfootball.com/participate/learn/brain-health/heading-in-football

[47] Bouvette V, Petit Y, De Beaumont L, Guay S, Vinet SA, Wagnac E (2024) American Football On-Field Head Impact Kinematics: Influence of Acceleration Signal Characteristics on Peak Maximal Principal Strain. Ann Biomed Eng 52(8):2134–2150. 10.1007/s10439-024-03514-z 38758459 10.1007/s10439-024-03514-z

[48] Liu Y, Domel AG, Cecchi NJ, Rice E, Callan AA, Raymond SJ, Zhou Z, Zhan X, Li Y, Zeineh MM, Grant GA, Camarillo DB (2021) Time Window of Head Impact Kinematics Measurement for Calculation of Brain Strain and Strain Rate in American Football. Ann Biomed Eng 49(10):2791–2804. 10.1007/s10439-021-02821-z 34231091 10.1007/s10439-021-02821-z

[49] Winter DA (2009) Biomechanics and Motor Control of Human Movement, 4th edn. Wiley, Hoboken, N.J. 10.1002/9780470549148

[50] Li Y, Mao H, Girshick R, He K (2022) Exploring Plain Vision Transformer Backbones for Object Detection. In: European Conference on Computer Vision, Tel Aviv, Israel. 10.48550/arXiv.2203.16527

[51] Peng D, Sun Z, Chen Z, Cai Z, Xie L, Jin L (2018) Detecting Heads Using Feature Refine Net and Cascaded Multi-Scale Architecture. arXiv. 10.48550/arXiv.1803.09256

[52] Varghese R, Sambath M (2024) YOLOv8: A Novel Object Detection Algorithm with Enhanced Performance and Robustness. In: 2024 International Conference on Advances in Data Engineering and Intelligent Computing Systems (ADICS), pp. 1–6. 10.1109/ADICS58448.2024.10533619

[53] Zhou Y, Gregson J (2020) WHENet: Real-time Fine-Grained Estimation for Wide Range Head Pose. arXiv. 10.48550/arXiv.2005.10353

[54] Everingham M, Van Gool L, Williams CKI, Winn J, Zisserman A (2010) The Pascal Visual Object Classes (VOC) Challenge. Int J Comput Vision 88(2):303–338. 10.1007/s11263-009-0275-4

[55] Valle R, Buenaposada JM, Baumela L (2021) Multi-task head pose estimation in-the-wild. IEEE Trans Pattern Anal Mach Intell 43(8):2874–2881. 10.1109/TPAMI.2020.3046323. arXiv:2202.02299 [cs] 33351746 10.1109/TPAMI.2020.3046323

[56] Cobo A, Valle R, Buenaposada JM, Baumela L (2024) On the representation and methodology for wide and short range head pose estimation. Pattern Recogn 149:110263. 10.1016/j.patcog.2024.110263

[57] Grove K, Karcher H, Ruh EA (1974) Jacobi fields and Finsler metrics on compact Lie groups with an application to differentiable pinching problems. Math Ann 211(1):7–21. 10.1007/BF01344138

[58] Karcher H (1977) Riemannian center of mass and mollifier smoothing. Commun Pure Appl Math 30(5):509–541. 10.1002/cpa.3160300502

[59] Manton JH (2004) A globally convergent numerical algorithm for computing the centre of mass on compact Lie groups. In: ICARCV 2004 8th Control, Automation, Robotics and Vision Conference, 2004., vol. 3, pp. 2211–2216. IEEE, Kunming, China. 10.1109/ICARCV.2004.1469774

[60] Lee J, Shin SY (2002) General construction of time-domain filters for orientation data. IEEE Trans Visual Comput Graphics 8(2):119–128. 10.1109/2945.998665

[61] Abate AF, Bisogni C, Castiglione A, Nappi M (2022) Head pose estimation: An extensive survey on recent techniques and applications. Pattern Recogn 127:108591. 10.1016/j.patcog.2022.108591

[62] Huynh DQ (2009) Metrics for 3D Rotations: Comparison and Analysis. Journal of Mathematical Imaging and Vision 35(2):155–164. 10.1007/s10851-009-0161-2

[63] Bland JM, Altman DG (1999) Measuring agreement in method comparison studies. Stat Methods Med Res 8(2):135–160. 10.1177/096228029900800204 10501650 10.1177/096228029900800204

[64] Li J, Bian S, Xu C, Chen Z, Yang L, Lu C (2023) HybrIK-X: Hybrid Analytical-Neural Inverse Kinematics for Whole-body Mesh Recovery. In: IEEE Transactions on Pattern Analysis and Machine Intelligence. arXiv. 10.48550/arXiv.2304.05690 10.1109/TPAMI.2025.3528979 40031204

[65] Stark NE-P, Henley ES, Reilly BA, Nowinski JS, Ferro GM, Madigan ML, Kuehl DR, Rowson S (2025) Uncalibrated Single-Camera View Video Tracking of Head Impact Speeds Using Model-Based Image Matching. Ann Biomed Eng 53(6):1359–1369. 10.1007/s10439-025-03705-2 40082330 10.1007/s10439-025-03705-2 PMC12075344

[66] Dunn M, Kennerley A, Murrell-Smith Z, Webster K, Middleton K, Wheat J (2023) Application of video frame interpolation to markerless, single-camera gait analysis. Sports Eng 26(1):22. 10.1007/s12283-023-00419-3

[67] Einfalt M, Ludwig K, Lienhart R (2023) Uplift and Upsample: Efficient 3D Human Pose Estimation with Uplifting Transformers. In: 2023 IEEE/CVF Winter Conference on Applications of Computer Vision (WACV), pp. 2902–2912. IEEE, Waikoloa, HI, USA. 10.1109/WACV56688.2023.00292

[68] Yuan Y, Iqbal U, Molchanov P, Kitani K, Kautz J (2022) GLAMR: Global Occlusion-Aware Human Mesh Recovery with Dynamic Cameras. In: Proceedings of the IEEE/CVF Conference on Computer Vision and Pattern Recognition, New Orleans, LA, USA. 10.48550/arXiv.2112.01524

[69] Jiang T, Billingham J, Müksch S, Zarate J, Evans N, Oswald MR, Pollefeys M, Hilliges O, Kaufmann M, Song J (2025) WorldPose: A World Cup Dataset for Global 3D Human Pose Estimation. arXiv. 10.48550/arXiv.2501.02771

